# Mycolic acids: deciphering and targeting the Achilles' heel of the tubercle bacillus

**DOI:** 10.1111/mmi.13101

**Published:** 2015-07-30

**Authors:** Vijayashankar Nataraj, Cristian Varela, Asma Javid, Albel Singh, Gurdyal S. Besra, Apoorva Bhatt

**Affiliations:** ^1^ School of Biosciences and Institute of Microbiology and InfectionUniversity of BirminghamEdgbastonBirminghamB15 2TTUK

## Abstract

Mycolic acids are unique long chain fatty acids found in the lipid‐rich cell walls of mycobacteria including the tubercle bacillus *M* *ycobacterium tuberculosis*. Essential for viability and virulence, enzymes involved in the biosynthesis of mycolic acids represent novel targets for drug development. This is particularly relevant to the impact on global health given the rise of multidrug resistant and extensively drug resistant strains of *M* *. tuberculosis*. In this review, we discuss recent advances in our understanding of how mycolic acid are synthesised, especially the potential role of specialised fatty acid synthase complexes. Also, we examine the role of a recently reported mycolic acid transporter MmpL3 with reference to several reports of the targeting of this transporter by diverse compounds with anti‐*M* *. tuberculosis* activity. Additionally, we consider recent findings that place mycolic acid biosynthesis in the context of the cell biology of the bacterium, *viz* its localisation and co‐ordination with the bacterial cytoskeleton, and its role beyond maintaining cell envelope integrity.

## Introduction

**Figure 1:**
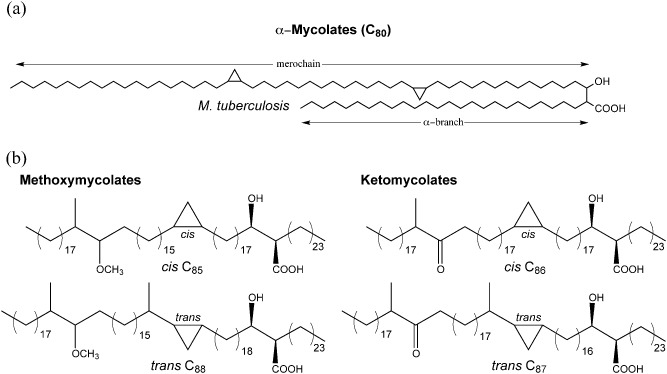
A. Fundamental structure of a mycolic acid shown using *M* *. tuberculosis* α‐mycolic acid as an example. B. Structures of other mycolic acid subclasses from *M* *. tuberculosis*.

The lipid‐rich cell wall of *Mycobacterium tuberculosis*, the bacterium that causes tuberculosis (TB), contains unique fatty acids termed mycolic acids (Minnikin and Polgar, 1966; Barry *et al*., 1998; Daffe and Draper, 1998). These long chain fatty acids, which have an alkyl side chain and a hydroxyl group at the α and β positions respectively (Fig. 1), are mainly found attached covalently to the distinctive peptidoglycan–arabinogalactan complex of the mycobacterial cell wall. Additionally, mycolic acids are constituents of outer cell envelope lipids including trehalose monomycolate (TMM), trehalose dimycolate (TDM) and glucose monomycolate, and they can also be found as free mycolic acids. Mycolic acids are derived from ‘housekeeping’ fatty acids, i.e. those important for fundamental cellular functions including cell membrane biosynthesis, which are synthesised by a ‘eukaryotic‐like’ modular type‐I fatty acid synthase termed FAS‐I (Bloch, 1977). Mycobacterial FAS‐I is bimodal and in addition to generating C_18_ fatty acids, *M. tuberculosis* FAS‐I also synthesises C_24–26_ fatty acids (Bloch, 1977). The former can also be fed into a second fatty acid synthase, the multienzyme, ‘prokaryotic‐like’ type‐II fatty acid synthase, FAS‐II, which eventually generates a long chain fatty acid termed the merochain. Malonyl‐CoA is the building block used by both FASs and its production requires acetyl‐CoA and an acetyl‐CoA carboxylase, AccD6 (Daniel *et al*., 2007). The malonate moiety from malonyl‐CoA is first transferred to an acyl carrier protein (ACP) by the enzyme FabD prior to entering the FAS‐II pathway for merochain biosynthesis (Kremer *et al*., 2001) via the proposed linking activity (so far only demonstrated *in vitro*) of the ketoacyl synthase FabH (Choi *et al*., 2000). FAS‐II consists of ‘core’ reductive cycle enzymes: a β‐ketoacyl‐ACP synthase, a β‐ketoacyl‐ACP‐reductase, a β‐hydroxyacyl‐ACP‐dehydratase and an enoyl‐ACP‐reductase that catalyse a reductive cycle using malonyl‐ACP as substrate to extend a growing acyl‐ACP chain by two carbons after each cycle, eventually yielding a merochain of chain length up to C_60_. Finally, the merochain is condensed with the FAS‐I derived C_24–26_ fatty acid by a polyketide synthase Pks13 (Gande *et al*., 2004; Portevin *et al*., 2004), to yield an oxo‐mycolic acid intermediate which is subsequently reduced by a mycolyl reductase to form a mature mycolic acid (Lea‐Smith *et al*., 2007; Bhatt *et al*., 2008). At some stage of this process, and it is not currently known when, unknown desaturase(s) introduce double bonds in the merochain, and subsequently modifying enzymes that include methyl transferases and cyclopropane synthases introduce modifications to the merochain resulting in the formation of different mycolic acid subclasses; *M. tuberculosis* produces three mycolic acid subclasses termed α, methoxy and keto mycolic acids (Fig. 1). The monomycolylated glycolipid TMM serves as both the carrier for mycolate transport (Grzegorzewicz *et al*., 2012; Varela *et al*., 2012), as well as the substrate for extracellular mycolyl transferases termed the Ag85 complex (Belisle *et al*., 1997; Puech *et al*., 2002). Enzymes of the Ag85 complex are responsible for the mycolylation of the arabinogalactan in the cell wall, as well as formation of TDM. While early research on mycolic acids was focussed on biochemical characterisation, the post genome sequencing era and the development of genetic tools for mycobacteria has resulted in a focus on identifying genes involved in the biosynthesis of these unique fatty acids. A vast majority of genes involved in mycolic acid biosynthesis are essential and thus cannot be studied via the generation of knockout strains. Though mycolic acids are unique, they are not exclusive to mycobacteria, but are also found in other related genera such as *Nocardia*, *Rhodococcus* and *Corynebacterium*. Three microbial species have particularly aided research into the genetic determinants of mycolic acid metabolism (Marrakchi *et al*., 2014). First, *Corynebacterium glutamicum*, by virtue of its ability to survive in the absence of mycolic acid production, has provided the opportunity to study viable mutants that lacked mycolic acids (Gande *et al*., 2004). Second, the fast growing saprophyte, *Mycobacterium smegmatis* has proven useful in the establishment of tools for generating conditional mutants, thus allowing us to study the loss of function of essential genes (Bhatt *et al*., 2005). *M. smegmatis* is also far more tolerant to mutations altering cell wall composition, which has been a particular advantage. And finally, the leprosy bacillus *Mycobacterium leprae*, which contains a ‘decayed’ genome, but produces mycolic acids, has aided the ‘bioinformatic filtering’ of candidate genes (Monot *et al*., 2005). However, some genes involved in mycolic acid biosynthesis are non‐essential in laboratory growth conditions and these are predominantly involved in merochain modification. *M. tuberculosis* mutants lacking these modifying enzymes have proved useful in outlining the importance of mycolic acids in immunomodulation, virulence and influencing host pathology (Dubnau *et al*., 2000; Glickman *et al*., 2000; Rao *et al*., 2005; 2006). Additionally, *M. tuberculosis* has two genes that each encode a β‐ketoacyl‐ACP synthase, a core FAS‐II enzyme. While one of these genes, *kasA*, is essential (Bhatt *et al*., 2005), the other, *kasB*, is not (Bhatt *et al*., 2007a). Interestingly, the *M. tuberculosis kasB* null mutant produces shorter mycolic acids, and while the mutant was severely attenuated, it was able to persist in mice without displaying any of the tissue pathology associated with infection (Bhatt *et al*., 2007a).

The essentiality of mycolic acids for viability and for virulence highlights the potential of components of the mycolic acid biosynthesis machinery as attractive drug targets. While isoniazid, the hallmark anti‐TB drug that targets the FAS‐II enoyl‐ACP‐reductase InhA has been in use for many years, mycolic acid biosynthesis pathways remain largely untapped as drug targets. This is especially timely and relevant given the rise of multidrug resistant and extensively drug resistant strains of *M. tuberculosis* (MDR‐TB and XDR‐TB), and more worryingly the relatively recent reports of ‘untreatable’ TB (Udwadia *et al*., 2012). While there have been numerous reviews on the cell wall of mycobacteria, very few have focussed exclusively on mycolic acids. Takayama and colleagues wrote the first comprehensive post‐genome sequence review that not only collated existing knowledge about mycobacterial mycolic acid biosynthesis but also formulated hypothesis about yet unidentified processes using bioinformatic searches (Takayama *et al*., 2005). The field has advanced rapidly since that review in 2005, and more recently Marrakchi *et al*. (2014) have offered a comprehensive review of these advances. The aim of this microreview is not to replicate the information contained in these earlier reviews. Instead we wish to reflect on advances made in the field since the latter half of 2013. We also consider the broader implications of these findings in terms of how they advance our understanding of mycolic acid biosynthesis in context of the biology of the bacterium, including higher order structures and integration with the cell biology of the bacterium.

## Fatty acids and hotdogs: finding the elusive dehydratase

Core components of a FAS‐II complex typically contain four enzymes constituting a reductive cycle that catalyzes the extension of an acyl‐ACP chain by two carbons. While three of the four components (KasA/KasB, MabA and InhA) for the merochain producing mycobacterial FAS‐II had been identified by the late 1990s, until late the dehydratase catalysing conversion of β‐hydroxyacyl‐ACP to enoyl‐ACP remained unidentified. The two operons harbouring the genes encoding the other three core FAS‐II components (InhA and MabA, and KasA/KasB) do not include genes with homology to FabZ or FabA, the classic dehydratase or dehydratase–isomerase enzymes found in FAS‐II complexes from other bacteria (Rock and Cronan, 1996). Furthermore, no *fabZ* or *fabA* homologues were found in the genome of *M. tuberculosis* H37Rv, suggesting that the dehydratase activity of FAS‐II in *M. tuberculosis* and other mycobacteria may be resulting from a dehydratase with a different or alternative enzymatic motif. A breakthrough came from a bioinformatic study that queried the role of 11 genes encoding putative *R*‐specific hydratase/dehydratase family, in the biosynthesis of mycolic acids (Castell *et al*., 2005). All contained a ‘hot dog’ fold that resembled the structure of FabA/Z, but contained a distinct catalytic site. Of these, only one gene, *Rv0636* was present in other mycolic acid producing genera and was demonstrated by Sacco *et al*. (2007) to be an essential gene and a dehydratase involved in mycolic acid biosynthesis. In a parallel study, Brown *et al*. (2007a, 2007b) used a drugs‐to‐target approach to demonstrate the role of *Rv0636* by showing that mycobacterial strains overexpressing *Rv0636* showed resistance to flavonoids known to target β‐hydroxyacyl‐ACP dehydratases in *Escherichia coli* and *Plasmodium*. Furthermore, the functional dehydratase component was shown to compose of a heterodimer of Rv0636 (HadB) with either Rv0635 (HadA) or Rv0637 (HadC). Both HadA and HadC also contain a hotdog fold and functional heterodimers thus have a ‘double hotdog’ fold. These asymmetric double hotdog folds are thought to play a dual role, with one (HadB) required for catalysis, and the other (from HadA or HadC) functioning as a stabiliser of long chain acyl groups that are typical of mycolic acids. This model has been lent further credence following the recently solved 3D structure of the HadA–HadB complex (Biswas *et al*., 2015). Furthermore, analogous to KasA and KasB (Kremer *et al*., 2002; Bhatt *et al*., 2007b), the two heterodimeric complexes HadA–HadB and HadB–HadC would be expected to differ in their specificities for different fatty acyl chain lengths, with the latter functioning to extend longer growing chains. Recently, Carrère‐Kremer *et al* (2015) demonstrated the presence of a second dehydratase, not found in *M. tuberculosis*, but present in non‐tuberculous mycobacteria (NTMs) including *M. smegmatis* (*MSMEG_6754*). The role of this non‐essential dehydratase, which is a ‘fused’ HadA–HadB‐like peptide, remains unclear but it was able to compensate for the loss of *hadB* in *M. smegmatis*. The authors were able to generate a mutant of *hadB* in *M. smegmatis* only when a chromosomally integrated, and constitutively expressed second copy of *MSMEG_6754* was present in the strain, indicating a functional redundancy with *hadB*. Interestingly, the recombinant strain did display an accumulation of shorter mycolic acid precursors, in addition to mycolic acid subspecies usually found in this fast growing species. However, the inability of this second dehydratase to rescue viability during attempts to generate a *hadB* knockout suggested a low level of functional protein encoded by *MSMEG_6754* was present in wild‐type *M. smegmatis* cells growing in laboratory conditions, and implied a regulatory mechanism controlling its expression. Indeed, *MSMEG_6754* was essential for survival in amoebae suggesting a specialised environmental role for this dehydratase in NTMs (Carrère‐Kremer *et al*., 2015).

## There are many FAS‐IIs

**Figure 2:**
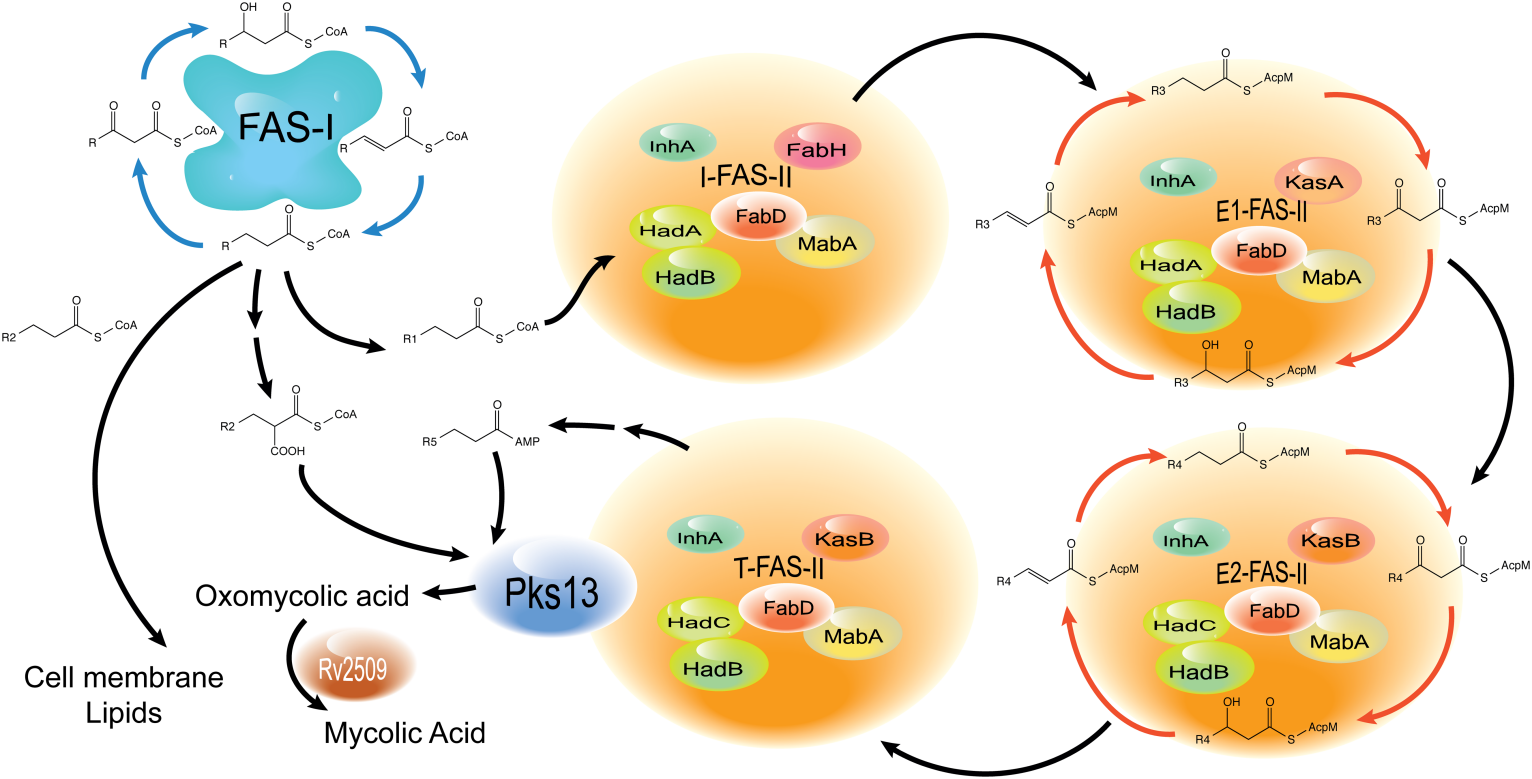
Schematic illustrating the biosynthesis pathway for mycolic acids in *M* *. tuberculosis* in the context of proposed specialised FAS‐II complexes. R1; C_16_–C_18_, R2; C_24_–C_26_, R3 represents a range of intermediate length meroacyl chains, R4 represents a range of longer meroacyl chains, R5 is the longest meroacyl chain. I‐FAS‐II; initiator FAS‐II, T‐FAS‐II; termination FAS‐II, and E1‐FAS‐II and E2‐FAS‐II represent elongation complexes.

Takayama *et al*. (2005) first suggested the presence of three FAS‐II ‘modules’ including a core elongation FAS‐II consisting of FabD, a β‐ketoacyl‐ACP synthase (KasA/KasB), MabA, InhA and, at the time unidentified, dehydratase component. Additionally, specialised FAS‐IIA and FAS‐IIB modules capable of elongation were hypothesised to be involved in the addition of *cis* unsaturations at distal and proximal position on the meromycolate chain respectively. The authors suggested that mycolic acid biosynthesis proceeds along linear ‘assembly line’ arrays similar to type‐II polyketide synthases, and that there are five such linear arrays, each terminating with a final condensation reaction catalysed Pks13. Each of these arrays, containing differing combinations of FAS‐II, FAS‐IIA, FAS‐IIB and modifying enzymes, was dedicated for the biosynthesis of one subclass of *M. tuberculosis* mycolic acids. The five arrays were thus involved in the biosynthesis of α mycolic acids, *cis*‐methoxy mycolic acids, *trans*‐methoxy mycolic acids, *cis*‐keto mycolic acids and *trans*‐keto mycolic acids. Experimental proof indicating the presence of such complexes came from protein–protein interaction studies conducted using the yeast two‐hybrid and three‐hybrid systems, and *in vitro* co‐immunoprecipitation studies using individual FAS‐II components (Veyron‐Churlet *et al*., 2004; 2005; Cantaloube *et al*., 2011). Cantaloube *et al*. (2011) extended earlier studies to define a ‘mycolic acid biosynthesis interactome’ consisting of different specialised FAS‐II elongation complexes, all of which contained a ‘core’ consisting of MabA, InhA and FabD (Fig. 2). An initiation FAS‐II (I‐FAS‐II) contains, in addition to the core, FabH and links FAS‐I to FAS‐II (Fig. 2). Two elongation complexes contain the core and either KasA and HadA–HadB (E1‐FAS‐II), or KasB and HadB–HadC (E2‐FAS‐II). E1‐FAS‐II is proposed to carry out the initial elongation cycles, with E2‐FAS‐II acting as unit for further elongation of meroacyl chains produced by E1‐FAS‐II (Fig. 2). Finally, a ‘termination’ FAS‐II complex involves Pks13 (Fig. 2), resulting in the Claisen condensation of a meromycolate chain with a FAS‐I derived C_26_ fatty acid to yield an oxo‐mycolic acid which is subsequently reduced by the mycolyl reductase encoded by *Rv2509*, to yield a mature mycolic acid moiety (Fig. 2). Furthermore, these studies have also shown that enzymes introducing modifications, such as methyltransferases, isomerases and cyclopropane synthases also interact with the elongation complex, suggesting that merochain modification occurs during elongation. Additionally, these modification enzymes show a preference for a particular type of Had heterodimer and consequently the type of elongation FAS‐II complex. CmaA2 and PcaA that introduce modifications to the proximal position of the merochain interact preferentially with HadBC, whereas MmaA3, the enzyme involved in the introduction of a methoxy group at the distal position, interacts preferentially with the HadA–HadB heterodimer.

## Mycolic acid transporters

While there has been considerable progress in the past two decades towards identifying genes involved in the biosynthesis of mycolic acids, mechanisms for their transport to the outside of the mycobacterial cell remained unknown. Takayama *et al*. (2005) had hypothesised that mycolic acids were likely transported in the form of TMM. The *M. tuberculosis* H37Rv genome encodes a class of membrane proteins termed mycobacterial membrane proteins large (MmpL), which are part of the resistance‐nodulation‐division (RND) family of efflux pumps (Cole *et al*., 1998; Domenech *et al*., 2005). Many of the 14 *mmpL* genes of *M. tuberculosis* are located adjacent to biosynthesis clusters for cell wall‐associated glycolipids, and have been shown to be transporters for the cognate lipids (Cox *et al*., 1999; Converse *et al*., 2003; Domenech *et al*., 2004; Belardinelli *et al*., 2014). We reckoned that TMM, also a glycolipid, was likely transported by a MmpL, and given the essentiality of mycolic acids for viability, the gene encoding this MmpL would be essential. Previous, unsuccessful attempts to generate a mutant of the gene *mmpL3* (*Rv0205c*) (Domenech *et al*., 2005), and its presence in the decayed *M. leprae* genome suggested that if an MmpL was involved in TMM transport, MmpL3 was the likely candidate for this function. Indeed we were able to demonstrate that *mmpL3* is essential in mycobacteria, and that conditional depletion of MmpL3 in *M. smegmatis* led to the intracellular accumulation of TMM (Varela *et al*., 2012). In parallel to our studies, Grzegorzewicz *et al* (2012) and Tahlan *et al*. (2012) identified MmpL3 as a target for novel anti‐TB drug candidates and subsequently demonstrated its role in as a translocator of TMM across the mycobacterial membrane. Whether MmpL3 is sufficient for the transport of TMM remains to be determined. However, recent findings from *C. glutamicum* indicate that transport of mycolates may be a complex process. Yamaryo‐Botte *et al*. (2015) showed that transient acetylation of the mycolyl moiety of trehalose monocorynomycolate (TMCM) was necessary for its subsequent transport across the corynebacterial membrane. Homologues of the TMCM acetyltransferase are present in mycobacteria, suggesting a similar process may occur in mycobacterial TMM transport.

Located in the same cluster as *mmpL3* is another *mmpL* gene, *mmpL11*, which was shown to be non‐essential (Domenech *et al*., 2005; Owens *et al*., 2013). However, a mutant of *mmpL11* in *M. smegmatis* was deficient in the transport of the less abundant monomeromycolyl diacylglycerol and a mycolate containing wax ester, and showed accumulation of MycPL (a proposed intracellular carrier of mycolic acids), indicating a role for MmpL11 in transport processes related to mycolic acid containing lipids (Pacheco *et al*., 2013). Interestingly both MmpL3 and MmpL11 have also been shown to play a role in heme transport, suggesting that they may have multiple roles in *M. tuberculosis* (Owens *et al*., 2013).

## Co‐ordination with polar growth

Mycobacteria, unlike other rod shaped bacteria such as *Bacillus*, exhibit polar growth where nascent peptidoglycan is synthesised and deposited at the poles during cell elongation (Kang *et al*., 2008). The tropomyosin‐like protein Wag31 (orthologue of *Bacillus* DivIVA) plays an important role in co‐ordinating polar growth; the protein localises to the tip of growing poles of mycobacterial cells and interacts with enzymes involved in cell wall biosynthesis (Kang *et al*., 2008; Jani *et al*., 2010). Two key papers have now shown that this interaction also involves components of the mycolate synthesising machinery. Carel *et al*. (2014) used GFP‐fusions of the FAS‐II components MabA, InhA and KasA to show that enzymes of the reductive cycle co‐localise with Wag31 at the ‘old’ growing poles. The authors also showed a fivefold enrichment of the mycolate transporter MmpL3 at the polar membranes. Meniche *et al*. (2014) took a different approach to identify additional components of the mycolic acid biosynthesis machinery that localise at the growing tips. Co‐purification experiments with endogenously tagged Wag31 and Pks13 in *M. smegmatis* revealed a polar complex consisting of AccA3, AccD4 and AccD5, members of an acyl‐CoA carboxylase (ACC) complex, Pks13 and FadD32, all enzymes required for the final stages of production of a nascent mycolic acid. The positions of the ACC components coincided with Wag31. However, Pks13 and FadD32 were found in a ‘subpolar’ location, suggesting that the Wag31‐associated complex and the terminal mycolate biosynthesis enzymes occupied exclusive regions of the polar tip, with new mycolic acids being deposited at a subpolar location in a growing tip.

## Beyond the wall

Given the abundance of mycolic acids in the lipid‐rich mycobacterial cell wall, it is tempting to assume that their role is limited to the structural integrity of the cell envelope. However, we now have a better understanding of the extended role played by mycolic acids, particularly TDM, in manipulating the host immune system and driving pathology during infection (reviewed extensively by Marrakchi *et al*., 2014). Mycolic acids have also been shown to play a role in the formation of biofilms often referred to as pellicular growth. The first link between pellicle formation and mycolic acids came from studies on a mutant strain of *M. smegmatis* that was deficient in the production of a GroEL1, a non‐essential chaperone in mycobacteria. The GroEL1 mutant was unable to form a pellicle on a liquid–air interface in a laboratory broth medium and displayed an altered mycolic acid profile (Ojha *et al*., 2005). Furthermore, GroEL1 was shown to directly interact with the FAS‐II enzyme, KasA. Mycolic acid composition also affects pellicle formation; a strain deficient in keto‐mycolic acid formation was unable to form pellicles (Sambandan *et al*., 2013). Furthermore, the *mmpL11* mutant of *M. smegmatis* that failed to transport monomeromycolyl diacylglycerol and a mycolate containing wax ester was defective in biofilm formation (Pacheco *et al*., 2013). Mycolates are not the only lipids to influence pellicle/biofilm formation; however, the enrichment of free mycolic acids in pellicles of mycobacteria showed that they play a significant role in the formation of this structure (Ojha *et al*., 2008).

## Targeting the Achilles' heel: developing drugs that inhibit mycolate metabolism

Unique components of bacteria that are also essential represent ideal drug targets as they allow development of drugs that specifically inhibit the aetiological agent of disease, while leaving the normal flora intact. In this regard, *M. tuberculosis* mycolic acids fulfil both these criteria. Indeed, the hallmark anti‐TB drug targets InhA, the core FAS‐II component (Banerjee *et al*., 1994). However, in the past 15 years a number of other inhibitors targeting other mycolate biosynthesis components have been described (reviewed by Marrakchi *et al*., 2014). These include compounds that target enzymes involved in mycolic acid condensation (Pks13, FadD32), and those involved in the biosynthesis/elongation of the fatty acyl chain (AccD5, AccD6, KasA/KasB, InhA, HadA, HadB, HadC, Ag85). Surprisingly, some targets have also included ‘non‐essential’ components; the compound dioctylamine inhibited multiple methyltransferases involved in mycolic acid modification, resulting in a loss of all mycolic acid cyclopropanation and cell death (Barkan *et al*., 2009). This was surprising as individual genes involved in mycolic acid cyclopropanation were not essential for growth, leading to the authors to suggest that loss of viability was likely due to the deleterious effect of loss of cyclopropanation on membrane fluidity. Similarly, thiacetazone was also shown to inhibit cyclopropanation of mycolic acids (Alahari *et al*., 2009). However, more recently, the focus of anti‐TB drug discovery has been on the TMM transporter, MmpL3. This is because spontaneous mutants resistant to a diverse group of compounds with anti‐*M. tuberculosis* activity were found to contain mutations in the *mmpL3* gene (Li *et al*., 2014). The first report was that of SQ109, a diamine, developed from the combinatorial library of ethambutol, which showed potent activity against drug‐sensitive and drug‐resistant mycobacteria (Protopopova *et al*., 2005). Subsequently, Tahlan *et al*. (2012) showed that SQ109 treatment led to TMM accumulation in *M. tuberculosis* and spontaneous SQ109‐resistant mutants contained single nucleotide polymorphisms (SNPs) in the *mmpL3* gene. Shortly thereafter, there were two reports of drugs targeting *M. tuberculosis*, the pyrrole derivative BM212 and the adamantly urea compound AU1235 (Grzegorzewicz *et al*., 2012; La Rosa *et al*., 2012). Mutants resistant to either compound were shown to harbour a mutated *mmpL3* allele. However, the divergent scaffolds from which the different inhibitors were derived, and their broad‐spectrum activity against non‐mycolate producing bacteria and fungal species, suggested that MmpL3 was probably not the direct target of these inhibitors. As mentioned earlier, MmpL3 and other mycobacterial MmpLs are members of the RND superfamily of proteins that require a proton motive force (PMF) for function. Li *et al*. (2014) have shown that SQ109 causes a dissipation of PMF in *M. tuberculosis*, thus disrupting MmpL3 function. Furthermore, exposure to SQ109 also led to reduction in lipid export by MmpL8 and MmpL10, indicating that effects of SQ109 were not MmpL3‐specific. This finding of a broader effect of SQ109 on multiple targets was at odds with the consistent isolation of resistant mutants with SNPs in *mmpL3*. The authors suggested that spontaneous mutants that are resistant to SQ109 (and other compounds) result from mutations in *mmpL3* as an early response to counteract toxic effects of these compounds (Li *et al*., 2014). Indeed these mutations mapped close to those predicted to participate in the proton gradient required for translocation of substrate. In light of several other reports of SNPs in *mmpL3* leading to resistance to diverse compounds, the idea that these are exclusively MmpL3 (and thus mycolic acid transport) targeting compounds needs to be revisited. The mycolic acid biosynthesis complex represents a unique target, one that is currently rendered unexploited in MDR‐TB and XDR‐TB strains (due to resistance to isoniazid). Further work on current known inhibitors of the enzymes mentioned earlier in this section will pave the way towards new inhibitors of mycolic acid biosynthesis.

## Future perspectives

A majority of the enzymatic components required for mycolic acid biosynthesis have now been identified; however, there are still some stages in mycolate metabolism that remain poorly understood. These include the process of introducing double bonds in merochains, and the enzymes involved in post‐Pks13 processing of mycolic acids that ultimately leads to the formation of TMM. However, in parallel, our understanding of mycolic acid biosynthesis and transport in context of a higher order of structures has been initiated by *in vitro* studies to detect interactions between FAS‐II components and other enzymes involved in the biosynthesis process. With the advance of microscopy techniques that enable visualisation of complexes in single bacterial cells, it is likely that such complexes may one day be demonstrated *in vivo*. Such studies could also indicate whether biosynthesis occurs at localised complexes that also integrate transport. A better understanding is also required of the co‐ordination of this process with polar growth, and with peptidoglycan and arabinogalactan deposition in the cell wall during cell division. These studies could potentially also open alternative avenues for targeting mycolic acid assembly via the design of complex‐disrupting inhibitors. Furthermore, the regulation of mycolic acid biosynthesis has only just begun to be unravelled. Regulation of activities of FAS‐II enzymes following post translation modification by Ser/Thr protein kinase‐mediated phosphorylation has been studied extensively *in vitro* (Veyron‐Churlet *et al*., 2009; Khan *et al*., 2010; Molle and Kremer, 2010; Molle *et al*., 2010; Slama *et al*., 2011; Corrales *et al*., 2012), and advances in ‘knock in’ methodologies have initiated studies to conduct the same *in vivo* (Vilcheze *et al*., 2014). While the transcriptional regulator MabR is known to regulate mycolic acid biosynthesis *in vitro* (Salzman *et al*., 2010), it is not known if transcription of merochain modification enzymes is regulated *in vivo*, resulting in changes to ratios of mycolic acid subclasses. In summary, this waxy ‘brick’ in the mycobacterial cell wall continues to be a focus of research on the tubercle bacillus, as we attempt to develop new and better therapies for this ancient disease.
